# Outdoor particulate matter exposure affects metabolome in chronic obstructive pulmonary disease: Preliminary study

**DOI:** 10.3389/fpubh.2023.1069906

**Published:** 2023-03-21

**Authors:** Tao Yu, Hanna Wu, Qingxia Huang, Fen Dong, Xuexin Li, Yushi Zhang, Ruirui Duan, Hongtao Niu, Ting Yang

**Affiliations:** ^1^Department of Pulmonary and Critical Care Medicine, Center of Respiratory Medicine, China-Japan Friendship Hospital, Beijing, China; ^2^National Center for Respiratory Medicine, Beijing, China; ^3^Institute of Respiratory Medicine, Chinese Academy of Medical Sciences, Beijing, China; ^4^National Clinical Research Center for Respiratory Diseases, Beijing, China; ^5^Peking Union Medical College, Chinese Academy of Medical Sciences, Beijing, China; ^6^Peking University China-Japan Friendship School of Clinical Medicine, Beijing, China; ^7^State Key Laboratory of Genetic Engineering, School of Life Sciences, Human Phenome Institute, Metabonomics and Systems Biology Laboratory at Shanghai International Centre for Molecular Phenomics, Fudan University, Shanghai, China

**Keywords:** air pollution, chronic obstructive pulmonary disease, metabolomics, PM_2.5_, arginine

## Abstract

**Introduction:**

The metabolomic changes caused by airborne fine particulate matter (PM_2.5_) exposure in patients with chronic obstructive pulmonary disease (COPD) remain unclear. The aim of this study was to determine whether it is possible to predict PM_2.5_-induced acute exacerbation of COPD (AECOPD) using metabolic markers.

**Methods:**

Thirty-eight patients with COPD diagnosed by the 2018 Global Initiative for Obstructive Lung Disease were selected and divided into high exposure and low exposure groups. Questionnaire data, clinical data, and peripheral blood data were collected from the patients. Targeted metabolomics using liquid chromatography-tandem mass spectrometry was performed on the plasma samples to investigate the metabolic differences between the two groups and its correlation with the risk of acute exacerbation.

**Results:**

Metabolomic analysis identified 311 metabolites in the plasma of patients with COPD, among which 21 metabolites showed significant changes between the two groups, involving seven pathways, including glycerophospholipid, alanine, aspartate, and glutamate metabolism. Among the 21 metabolites, arginine and glycochenodeoxycholic acid were positively associated with AECOPD during the three months of follow-up, with an area under the curve of 72.50% and 67.14%, respectively.

**Discussion:**

PM_2.5_ exposure can lead to changes in multiple metabolic pathways that contribute to the development of AECOPD, and arginine is a bridge between PM_2.5_ exposure and AECOPD.

## 1. Introduction

Chronic obstructive pulmonary disease (COPD) is a common chronic disease, mainly characterized by persistent respiratory symptoms and airflow restriction, resulting in airway and/or alveolar abnormalities. Its pathogenesis is not clear and is generally considered to be related to exposure to toxic air pollutants, especially particulate matters (1). The 2017 Global Burden of Disease Study showed that approximately 300 million individuals worldwide suffer from COPD, and the number of deaths due to COPD is as high as 3.2 million every year (2, 3), ranking fifth in the world's burden of disease. It is expected that COPD will become the third leading cause of death in the world by 2022 (4). The pathogenesis of COPD is very complex, and its potential cellular and molecular mechanisms remain unclear. COPD is a heterogeneous disease, which brings many challenges in its diagnosis, prognosis, and management. Therefore, it is imperative to identify reliable, easily measurable, and clinically relevant biomarkers of COPD to improve the prognosis of patients.

Among the pathogenic factors of COPD, particulate matter (PM) is closely related to the occurrence and development of COPD, especially PM with particle diameter < 2.5 μm (PM_2.5_), the main pathogenic component of air pollutants. Due to the small particle diameter, PM_2.5_ can be deposited in the alveoli, which leads to disorders of the airway immune system. PM_2.5_ enhances lung inflammation and enters blood circulation through the blood-gas barrier, causing serious health hazards to the body (5). Exposure to PM_2.5_ in the air is significantly associated with an increase in COPD prevalence and an increase in the acute exacerbation risk, hospitalization rate, and mortality of patients with COPD (6, 7). Concurrently, exposure to PM_2.5_ for a short time could lead to a systemic inflammatory response in patients with COPD (8). Therefore, it is important to explore the mechanism of PM_2.5_ exposures leading to COPD.

Metabolomics is the quantitative description of metabolites in organisms and their responses to changes in physiology, disease, and environment. It can provide mechanistic clues regarding biological processes and functions, which help deepen the understanding of the occurrence and development of diseases. It has been shown that disorders of phospholipid metabolism are associated with the development of emphysema and acute exacerbation of COPD (AECOPD) (9). Meanwhile, air pollutants are known to have an effect on metabolic disorders (10). Sun et al. showed that air pollution exposure can affect lipid metabolism disorders and ultimately lead to the development of diabetes mellitus (11). Air pollutants can affect arginine and proline metabolism, which can affect the cardiopulmonary system (12). Air pollutants can also promote atherosclerotic lesions by affecting cholesterol dysregulation (13). However, limited studies have examined the effects of air pollution on the development of COPD.

Therefore, the present study aims to recognize which metabolites and metabolic pathways are influenced by outdoor air pollutants and the predictive value of the metabolites on AECOPD. We divided the patients with COPD into high and low pollutant exposure groups and explored the effect of PM_2.5_ on metabolism by performing targeted metabolomic assays on plasma to identify relevant markers that can predict AECOPD due to air pollution.

## 2. Methods

### 2.1. Study design and participants

This was an observational clinical study conducted from January 2018 to March 2019. The participants were patients with COPD in the Beijing-Tianjin-Hebei region, China. The inclusion criteria were as follows: (1) age 45–75 years; (2) met the diagnostic criteria for the global initiative for COPD (GOLD-2018) and were in a stable stage of disease within 1 month at the time of enrollment; (3) resided in the local area continuously for more than 2 years, with no long-term plan to leave the area during the survey period; and (4) had no smoking history or had stopped smoking for more than half a year to rule out the effect of smoking on AECOPD. The exclusion criteria were as follows: (1) had complications, such as malignant tumor, severe cardiovascular and cerebrovascular disease, liver and kidney insufficiency, and active pulmonary tuberculosis; (2) received efficacy evaluation for epilepsy or mental illness; (3) underwent chest, abdominal or ophthalmic surgery in the past 3 months; and (4) pregnant and lactating women. All patients were surveyed using a questionnaire that included the basic information on the COPD assessment test (CAT), modified Medical Research Council (mMRC) dyspnea scale, St. George's respiratory questionnaire, pulmonary function examination, physical examination (height, weight, and blood pressure), and peripheral blood collection. After 3 months, telephone follow-up was conducted to monitor any acute exacerbations during this period, including requiring treatment with corticosteroids and/or antibiotics or hospitalization (9). The research protocol was approved by the ethics committee of the China-Japan Friendship Hospital, and all participants signed informed consent (IRB Approval Number: 2017–19). All methods were carried out in accordance with the Declaration of Helsinki.

### 2.2. Outdoor PM_2.5_ exposure assessment

The outdoor PM_2.5_ exposure concentrations in this study were obtained from the China National Environmental Monitoring Center (http://www.cnemc.cn/), and the daily estimates of pollutants at each monitoring station were calculated as the 24-hour average concentrations at the corresponding monitoring station. After matching the PM_2.5_ data with the subjects' addresses from the questionnaires, the average concentration of PM_2.5_ for the month before the survey date in the participant's city was obtained. The participants were divided into a high exposure group (>75 μg/m^3^) and a low exposure group (< 35 μg/m^3^) based on the PM_2.5_ concentrations according to the National Ambient Air Quality Standards (GB 3095-2012).

### 2.3. Plasma-targeted metabolomic assays

Blood samples were collected from all patients in a fasting state and transferred to the Department of Laboratory Medicine of China-Japan Friendship Hospital and stored at −80°C for metabolomic analysis.

Targeted quantitative metabolomics analysis was performed using the Biocrates P500 platform (Biocrates Life Sciences AG, Innsbruck, Austria). Using a previously described method, 10 μL of each plasma sample was pretreated in 96-well plates (14) and then analyzed using liquid chromatography-tandem mass spectrometry (LC-MS) and flow injection analysis (FIA). LC-MS used two liquid chromatography separation methods, and mass spectrometry used the multiple reaction monitoring mode, collecting positive and negative ion modes, respectively. FIA used the same liquid chromatography separation method and collected data by two mass spectrometry methods in the positive ion mode. The raw mass spectrometry data were analyzed using the MetLIMS software (Biocrates Life Sciences AG), and the concentration of the metabolites in the samples was calculated using the 7-point calibration standard curve method and single-point method for LC-MS and FIA data, respectively. All samples were randomly assigned, and quality control (QC) samples with known concentrations were inserted into the sample cohort to assess the reproducibility of the data. The filter thresholds for metabolites were QC samples with coefficients of variation ≥ 25% and missing values ≥ 30%. The metabolites were annotated via the Human Metabolite Database (HMDB).

### 2.4. Statistical analysis

The differences in the demographic characteristics between the groups were analyzed using *t*-tests for continuous variables and chi-square tests for categorical variables. For metabolomic analysis, the Mann–Whitney non-parametric test and orthogonal partial least squares discriminant analysis (OPLS-DA) were used to analyze between-group differences in metabolites after metabolite concentrations were log-transformed. *P*-value < 0.01 and variable importance in the projection score (VIP) > 1.5 were used as the threshold for differential metabolite screening. Pathway analysis was performed for significantly changed metabolites with HMDB numbers using the Kyoto Encyclopedia of Genes and Genomes (KEGG; Kanehisa Laboratories, Kyoto, Japan) database, and enrichment analysis was performed using the Small Molecule Pathway Database. Logistic regression was used to assess the association of variables with the risk of acute exacerbation during the 3 months of follow-up, with sex, age, smoking history, and BMI as covariates. The predictive power of important metabolites was assessed using receiver operating characteristic (ROC) curves and the area under the curve (AUC). Data and statistical analyses were performed using SPSS 26.0 (IBM, Armonk, NY, USA). OPLS-DA was performed using SIMCA 14.0 (Umetrics, Umeå, Sweden). Pathway analyses were performed on the platform MetaboAnalyst 5.0 (https://www.metaboanalyst.ca). Logistics regression was calculated by SPSS 26.0 (IBM, Armonk, NY), and ROC curves were drawn by GraphPad Prism 9.0. A *P*-value < 0.05 was considered statistically significant.

## 3. Results

### 3.1. Characteristics of the participants

Thirty-eight participants with complete data were included in the analysis, with 19 in the high exposure group (mean age 62.8 ± 7.8 years, 84.2% male) and 19 in the low exposure group (mean age 62.7 ± 4.7 years, 63.2% male). The demographic characteristics of the study population are shown in Table 1. The average concentration of outdoor PM_2.5_ exposure was 19.0 ± 1.02 μg/m^3^ and 106.0 ± 15.3 μg/m^3^ in the low and high exposure groups, respectively. The presence or absence of AECOPD during the 3 months of follow-up and CAT questionnaire scores were statistically different between the two groups (*P* = 0.02, 0.01, and 0.008, respectively).

**Table 1:** Characteristics of the study participants.

**Variables**	**Low exposure**	**High exposure**	***P*-value**
Sex (male, %)	12.00 (63.20%)	16.00 (84.20%)	0.14
Age (years)	62.74 ± 4.74	62.84 ± 7.77	0.96
BMI (kg/m^2^)	26.00 ± 5.50	24.49 ± 5.00	0.40
Smoking history (*n*, %)	9.00 (47.40%)	14.00 (73.70%)	9.70 × 10^−2^
AECOPD during the 3 months of follow-up (*n*, %)	2.00 (10.50%)	8.00 (42.10%)	2.70 × 10^−2*^
FEV_1_%pred (%)	53.69 ± 12.72	46.47 ± 16.59	0.19
FVC (L)	2.65 ± 0.78	2.64 ± 0.68	0.88
FEV_1_/FVC (%)	54.46 ± 8.60	50.70 ± 8.47	8.19 × 10^−2^
DL_CO_ (ml·kPa/s)	6.44 ± 2.10	8.58 ± 7.00	0.23
FeNO (ppb)	27.53 ± 19.85	29.47 ± 23.20	0.80
Systolic BP (mm Hg)	123.67 ± 11.50	126.22 ± 15.38	0.58
Diastolic BP (mm Hg)	78.23 ± 11.45	76.93 ± 8.31	0.70
SGRQ, total score	29.50 ± 11.86	36.01 ± 20.27	0.20
mMRC, total score	1.11 ± 0. 79	1.7 ± 1.16	6.44 × 10^−2^
mMRC score ≥1 (*n*, %)	15 (78.90%)	16 (84.20%)	0.68
CAT, total score	10.37 ± 8.46	15.58 ± 7.90	6.42 × 10^−2^
CAT score ≥10 (*n*, %)	7.00 (36.80%)	15.00 (78.90%)	8.58 × 10^−3*^
Diabetes	3 (15.79%)	3 (15.79%)	1
Outdoor PM_2.5_ concentration (μg/m^3^)	19.02 ± 1.02	106.00 ± 15.29	8.70 × 10^−24*^

AECOPD, acute exacerbation of chronic obstructive pulmonary disease; BMI, body mass index; BP, blood pressure; CAT, COPD assessment test; CRP, C-reactive protein; DL_CO_, diffusion capacity of carbon monoxide; FeNO, Exhaled nitric oxide; FEV_1_%pred, forced expiratory volume in the first second predicted; FEV_1_/FVC, forced expiratory volume in 1 s to forced vital capacity ratio; mMRC, Modified Medical Research Council; SGRQ, St George's Respiratory Questionnaire.

Data are presented as the mean ± standard deviation.

^*^P < 0.05.

### 3.2. Effects of PM_2.5_ exposure on metabolites

**Figure 1:**
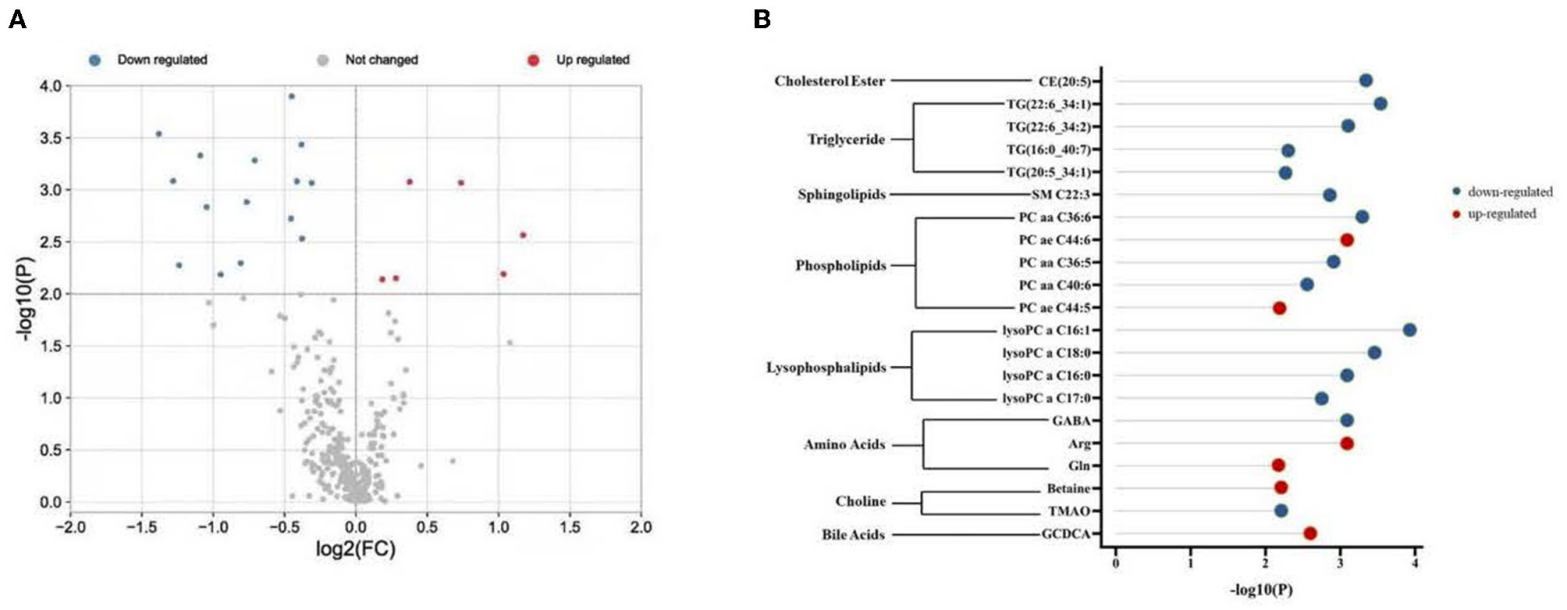
Different metabolites in two groups. **(A)** Volcano plot shows -log_10_ P against the log_2_-fold change of 311 metabolites in the high exposure group vs. the low exposure group. Blue denotes down-regulated metabolites, red denotes up-regulated metabolites, and gray denotes no change. **(B)** Twenty-one differentially regulated metabolites were associated with PM_2.5_ exposure. Red indicates up-regulation and blue indicates down-regulation. PM_2.5_, PM with particle diameter <2.5 μm; GABA, γ-aminobutyric acid; Arg, arginine; Gln, Glutamine; TMAO, trimethylamine N-oxide; GCDCA, glycochenodeoxycholic acid.

**Figure 2:**
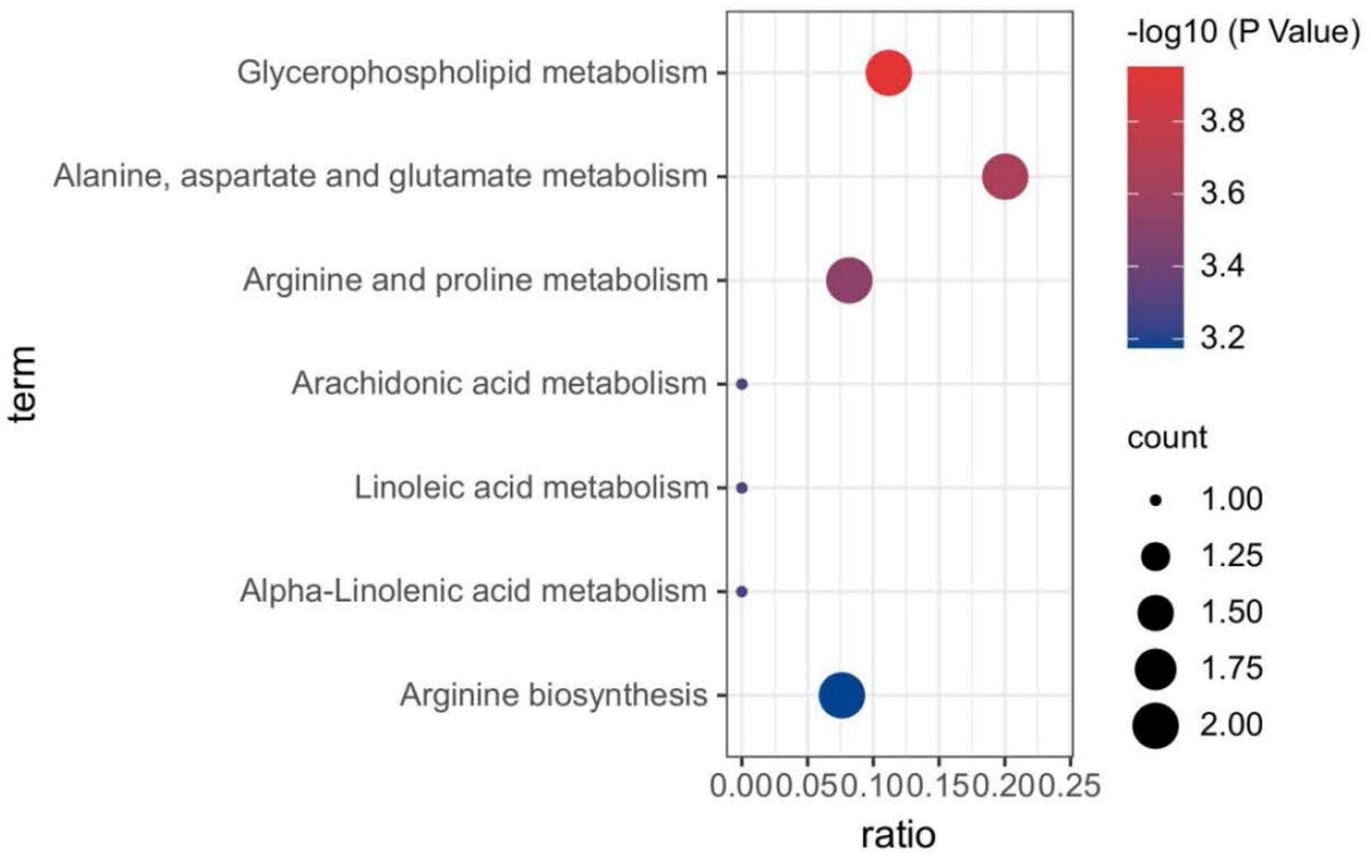
Biological pathways associated with PM_2.5_ exposure. The color gradient indicates the significance of the pathway ranked by *P*-value (blue: higher *P*-values and red: lower *P*-values), and circle size indicates the pathway impact score (the larger circle the higher impact score). PM_2.5_, PM with particle diameter <2.5 μm.

**Figure 3:**
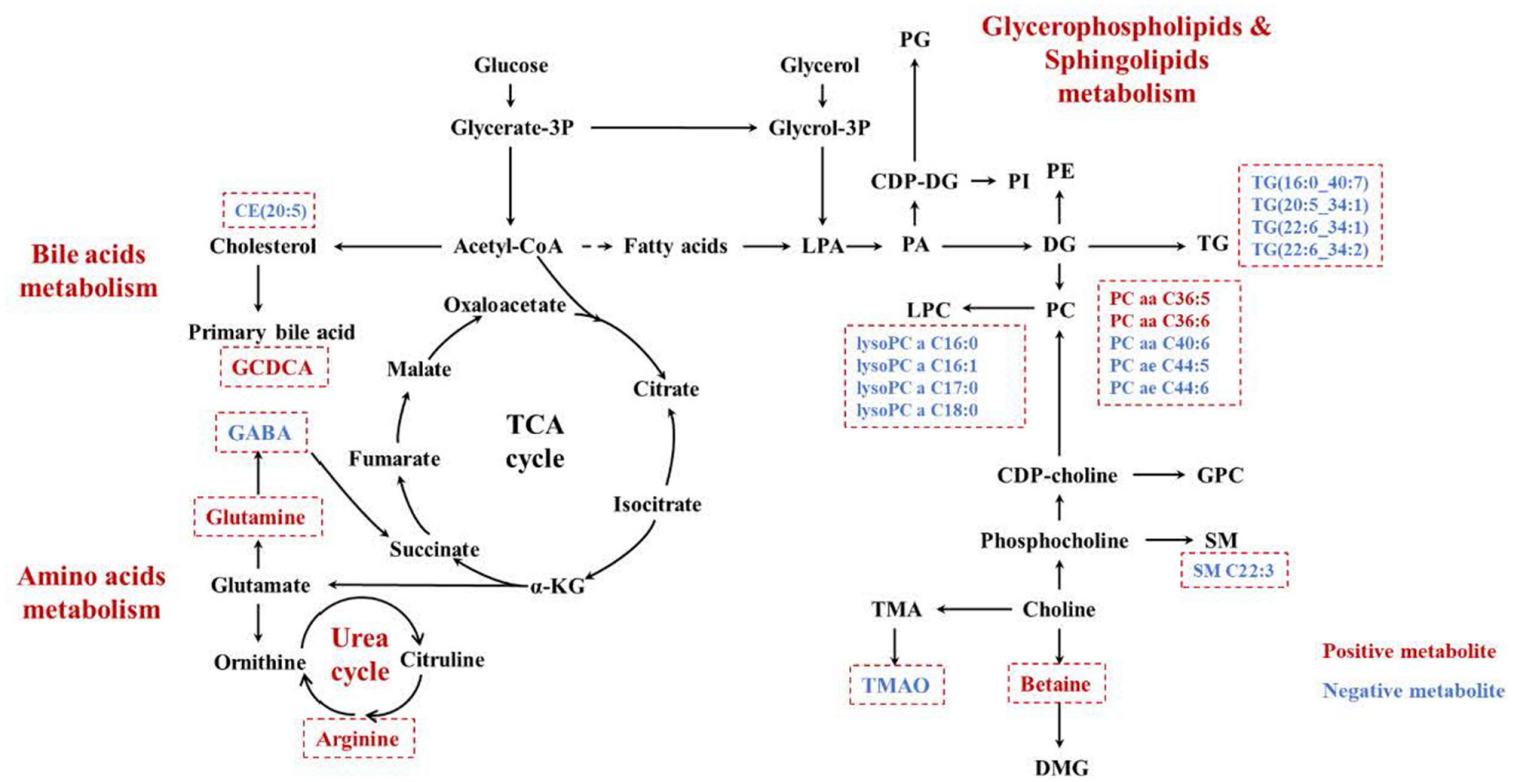
The increased metabolites were marked in red, while the decreased ones were marked in blue. PG, polygalacturonase; CDP-DG, cytidine 5'-diphosphate 1,2-diacyl-sn-glycerol; LPA, lysophosphatidic acid; PA, phosphatidic acid; PI, phosphoinositide; PE, phosphatidylethanolamine; DG, diacylglycerol; PC, phosphatidylcholine; LPC, lysophosphatidylcholine; TG, triglyceride; GPC, l-α- Glycerophosphatidylcholine; SM, sphingomyelin; DMG, dimethylglycine; TMA, trimethylamine; TMAO, trimethylamine N-oxide; PM_2.5_, PM with particle diameter <2.5 μm.

Plasma metabolomics data from the 38 participants were collected. The LC-MS method and FIA method were used to obtain 106 and 524 metabolites, respectively. Thus, 311 metabolites were included in the analysis after data QC. OPLS-DA analysis showed a separation trend between the low- and high-exposure groups. Twenty-one metabolites were statistically different between the two groups based on a *P*-value < 0.01 and VIP >1.5 (Figure 1A and Table 2, see also Supplementary material 1). These metabolites included cholesterol ester, triglycerides, sphingolipids, phospholipids, lyso-phospholipids, amino acids, choline, and bile acids. The volcano plot and heat map showed that six metabolites showed elevated expression, and the expression levels of 15 metabolites decreased with exposure to high PM_2.5_ levels compared with exposure to low PM_2.5_ levels (Figure 1, see also Supplementary material 2). Although the inclusion criteria are patients who had no smoking history or had stopped smoking for more than half a year to rule out the influence of smoking, we also divided the patients into two groups according to smoking status (ever smoker vs. never smoker), and compared the different metabolites after exposure to high and low PM_2.5_ level (Supplementary material 3). The results showed that, in both groups, most metabolites have certain statistical significance. This means that air pollution exposure had a certain impact on ever smoker and never smoker. To further investigate the effects of molecular pathways underlying PM_2.5_ exposure and AECOPD, we performed pathway analysis based on the KEGG database (Figure 2). It was observed that seven metabolic pathways were correlated with PM_2.5_ exposure, including glycerophospholipid, alanine, aspartate, glutamate, arginine, proline metabolism, and arginine biosynthesis. These results suggest that metabolic disturbances occur after PM_2.5_ exposure, including disturbances in amino acid, bile acid, and lipid metabolisms (Figure 3).

**Table 2:** Twenty-one differential metabolites in the two groups.

**Sample identification**	**FC (high/low)**	***P*-value**	**VIP score**
lysoPC a C16:1	0.74	1.27 × 10^−4^	2.62
TG (22:6_34:1)	0.38	2.90 × 10^−4^	2.18
lysoPC a C18:0	0.77	3.68 × 10^−4^	2.53
CE (20:5)	0.47	4.67 × 10^−4^	2.37
PC aa C36:6	0.60	5.22 × 10^−4^	2.39
TG (22:6_34:2)	0.41	8.27 × 10^−4^	1.93
GABA	0.74	8.31 × 10^−4^	2.27
PC ae C44:6	1.30	8.40 × 10^−4^	2.40
Arginine	1.67	8.58 × 10^−4^	2.56
lysoPC a C16:0	0.81	8.61 × 10^−4^	2.33
PC aa C36:5	0.59	1.31 × 10^−3^	2.41
SM C22:3	0.47	1.47 × 10^−3^	2.28
lysoPC a C17:0	7.01 × 10^−3^	1.89 × 10^−3^	2.21
GCDCA	2.25	2.73 × 10^−3^	2.44
PC aa C40:6	0.77	2.95 × 10^−3^	2.31
TG (16:0_40:7)	0.54	5.08 × 10^−3^	1.98
TG (20:5_34:1)	0.47	5.32 × 10^−3^	2.48
Betaine	2.05	6.46 × 10^−3^	1.76
TMAO	0.52	6.53 × 10^−3^	2.00
PC ae C44:5	1.22	7.09 × 10^−3^	1.93
Glutamine	1.14	7.27 × 10^−3^	2.01

FC, fold change; VIP, Variable importance in the projection; GABA, γ-aminobutyric acid; GCDCA, glycochenodeoxycholic acid; TMAO, trimethylamine N-oxide.

### 3.3. Predicting the risk of AECOPD based on metabolic changes

**Figure 4:**
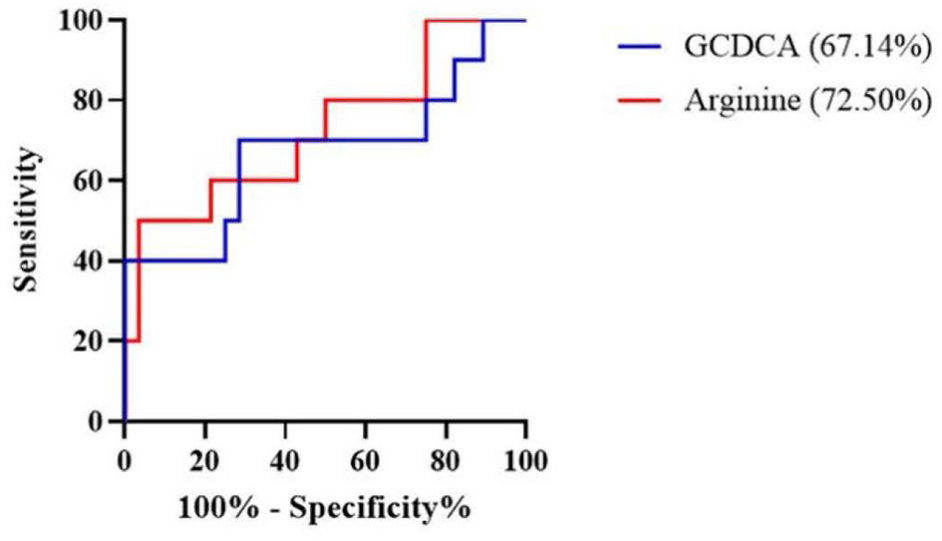
Receiver operating characteristic (ROC) curve revealed candidate biomarkers for predicting AECOPD. AECOPD, acute exacerbation of chronic obstructive pulmonary disease; GCDCA, glycochenodeoxycholic acid.

The association of 21 metabolites with the risk of AECOPD in the next 3 months is shown in Table 3. Arginine and glycochenodeoxycholic acid (GCDCA) were positively associated with AECOPD when corrected for sex, age, BMI, and smoking history (OR = 1.028, 95%CI 1.001–1.056, *P* = 0.045 and OR = 3.974, 95%CI 1.017–15.537, *P* = 0.047, respectively). Subsequently, the AUC obtained from the ROC analysis was used to assess the predictive performance of these two metabolites for AECOPD (Figure 4). Arginine had better predictive performance (AUC = 72.50%, 95% CI 0.53–0.92) compared with that of GCDCA (AUC = 67.14%, 95% CI 0.45–0.90).

**Table 3:** Relationship between metabolites and AECOPD during the 3 months of follow-up.

**Metabolites**	**Acute exacerbation of COPD**	
	**OR (95%CI)** ^**a**^	***P* ^a^**
LysoPC a C16:1	0.512 (0.120–2.179)	0.365
TG (22:6_34:1)	0.967 (0.812–1.153)	0.709
LysoPC a C18:0	0.996 (0.906–1.095)	0.937
CE (20:5)	1.004 (0.956–1.056)	0.861
PC aa C36:6	5.522 (0.064–477.666)	0.453
TG (22:6_34:2)	0.977 (0.851–1.120)	0.735
GABA	0.040 (0.001–2088767.376)	0.723
PC ae C44:6	0.693 (0.042–11.346)	0.797
Arginine	1.028 (1.001–1.056)	0.045^*^
LysoPC a C16:0	0.996 (0.961–1.032)	0.813
PC aa C36:5	1.048 (0.881–1.246)	0.596
SM C22:3	3.577 (0.002–6960.684)	0.742
LysoPC a C17:0	2.514 (0.431–14.667)	0.306
GCDCA	3.974 (1.017–15.537)	0.047^*^
PC aa C40:6	1.093 (0.875–1.366)	0.434
TG (16:0_40:7)	0.872 (0.496–1.530)	0.632
TG (20:5_34:1)	0.982 (0.582–1.658)	0.947
Betaine	1.025 (0.864–1.215)	0.778
TMAO	0.785 (0.471–1.307)	0.351
PC ae C44:5	1.031 (0.030–35.072)	0.987
Glutamine	1.001 (0.993–1.009)	0.814

^a^Adjusted for sex, age, BMI, smoking history.

^*^P < 0.05.

AECOPD, acute exacerbation of chronic obstructive pulmonary disease; BMI, body mass index; COPD, chronic obstructive pulmonary disease; GABA, γ-aminobutyric acid; GCDCA, glycochenodeoxycholic acid; TMAO, trimethylamine N-oxide.

## 4. Discussion

PM_2.5_ exposure can reportedly lead to acute exacerbation in patients with COPD, but the exact mechanism of action is unclear. In this study, we divided the patients into high and low exposure groups according to the exposure level of outdoor PM_2.5_, and detected the plasma metabolites. We found that 21 metabolites showed significant changes between the two groups. Meanwhile, we found that PM_2.5_ exposure could affect lipid-, amino acid-, and bile acid-related metabolism (Figure 3) by performing targeted metabolomic assays on the plasma of patients with COPD with different PM_2.5_ exposure levels. Correlation analysis of the metabolites with the occurrence of acute exacerbations in 3 months revealed that arginine and GCDCA were positively correlated with the occurrence of AECOPD, while arginine better predicted AECOPD. These findings suggest that arginine is a potential marker of PM_2.5_-induced AECOPD.

Several studies have confirmed the correlation between PM_2.5_ and the development of COPD. Yang et al. found that long-term PM_2.5_ exposure can reduce the airway and small airway function and impair lung function (15). Liu et al. conducted a 16-year cohort study and found that for every 5 μg/m^3^ increase in PM_2.5_ concentration, the adjusted hazard ratio for associations with COPD incidence was 1.17 (16). The hospitalization and mortality rates for COPD increase during periods of high air pollution. Each 10 μg/m^3^ increase in PM_2.5_ is associated with a 1.61% increase in the risk of hospitalization for patients with COPD in the United States (17) and 0.82% in Beijing (18). In a meta-analysis, Chen et al. summarized the effect of air pollution exposure on COPD mortality and showed that a 10 μg/m^3^ increase in PM_2.5_ was associated with a 1.11% increase in the risk of COPD-related death (19).

Chronic exposure to air pollution can lead to oxidative stress and inflammatory responses in the body. Air pollutants may act directly as oxidants or generate free radicals, causing oxidative stress (20). The glycerolphospholipid metabolism found in this study may be associated with this oxidative stress response. Air pollutants activate phospholipase A2, which hydrolyzes phospholipids in the cell membrane, thereby disrupting the alveolar structure (21, 22). This result is consistent with the results of the study by Nassan et al. (23). In addition to glycerolphospholipids metabolism, we found that PM_2.5_ exposure was associated with bile acid metabolism. An intermediate in the biosynthesis pathway of bile acid, 27-Hydroxycholesterol (27-OHC), was significantly enhanced in the lung tissues of patients with COPD. Meanwhile, amino acid metabolism, including alanine, aspartate, glutamate, arginine, and proline metabolism and arginine biosynthesis were also affected by PM_2.5_ exposure. Similarly, a study in 2018 showed elevated expression of amino acid metabolites in the blood of participants from high pollution exposure areas close to roadways, including arginine, histidine, γ-linolenic acid, and hypoxanthine (24). Meanwhile, a 2021 analysis of the effects of air pollution exposure on amino acid metabolites in the plasma of participants using a lagged effects model showed that exposure to PM_2.5_ was significantly and positively associated with elevated asparagine and glutamine over a 24-h period (25). These results suggest that amino acid metabolism plays an important role in PM_2.5_ pathogenesis.

On performing logistic regression of metabolites screened for association with PM_2.5_ exposure and AECOPD in the next 3 months, we found that arginine and GCDCA were positively associated with AECOPD in controlling sex, age, BMI, and smoking history, while ROC curves indicated that arginine better predicted AECOPD. This is similar to the findings of a previous study, which found that arginine expression was elevated in the plasma of patients with COPD compared with that in the healthy population and was further elevated in AECOPD (26). Meanwhile, Grasemann et al. showed that the arginine content in sputum was negatively correlated with FEV_1_ (27). Arginine is a semi-essential amino acid that is an important substrate for enzymes such as nitric oxide synthase (NOS) and arginase (28). Arginase converts arginine to ornithine and urea, while NOS converts arginine to citrulline with the production of NO. Exhaled NO is associated with airway inflammation, and ornithine is a precursor of proline and polyamines (e.g., putrescine, spermidine, and spermidine), which promote collagen production and cell proliferation (29, 30). In addition, asymmetric dimethylarginine (ADMA) is an endogenous competitive inhibitor of NOS, produced by arginine (31). ADMA is evolving as a marker of cardiovascular risk and has been studied in allergic asthma models (32), obesity-induced asthma studies (33, 34), obstructive sleep apnea syndrome studies, and COPD studies (35). Therefore, an increase in arginine concentration will lead to an increase in the expression of NOS and ADMA, which has a potential effect on airway inflammation and airway remodeling.

The present study has some limitations. First, the PM_2.5_ exposure data obtained in this study were from atmospheric monitoring stations, and no indoor air pollution levels were detected, which is not a good representation of the true PM_2.5_ exposure levels of patients. In addition, this study only explored the effects of PM_2.5_ on COPD patients and did not investigate the effects of other pollutants on metabolites, which has some limitations. Second, we did not consider indicators related to the patients' diet, exercise, sleep, and region that may affect metabolism. Third, the information about AECOPD was obtained from the patient's memories, and the questionnaire and blood samples were not tested when the patients were in acute exacerbation. In the future study, patients will be evaluated for acute exacerbation, so as to determine the impact of PM on AECOPD. Moreover, this study had a small sample size, restricting the generalizability of our findings. Further large sample studies are needed to detect metabolites in sputum or bronchoalveolar lavage fluid and conduct related mechanism studies to further explore the effect of air pollution on metabolites and the mechanism of AECOPD.

## 5. Conclusions

In summary, this study showed that PM_2.5_ exposure can lead to changes in multiple metabolic pathways that contribute to the development of AECOPD using metabolomic assays in plasma from patients with COPD subjected to high and low exposure levels. Our results suggest that arginine is a bridge between PM_2.5_ exposure and AECOPD; future studies can further explore the mechanism of elevated arginine expression caused by PM_2.5_ exposure and the correlation between arginine and AECOPD. Simultaneously, reducing PM_2.5_ concentration and going out in polluted weather are essential measures to prevent AECOPD.

## Data availability statement

The raw data supporting the conclusions of this article will be made available by the authors, without undue reservation.

## Ethics statement

The studies involving human participants were reviewed and approved by the Ethics Committee of the China-Japan Friendship Hospital. The patients/participants provided their written informed consent to participate in this study.

## Author contributions

The study was proposed and the manuscript was revised by TiY. Material preparation and data collection were performed by TaY, HN, XL, and RD. Data analysis was performed by TaY, HW, FD, and QH. The first draft of the manuscript was written by TaY and HW. All authors commented on subsequent versions of the manuscript, read, approved the final manuscript, and contributed to the study conception and design.
