# Supplementary material for: Outdoor particulate matter exposure affects metabolome in chronic obstructive pulmonary disease: Preliminary study
Source: Front Public Health. 2023 Mar 21;11:1069906. doi: 10.3389/fpubh.2023.1069906 (PMC10070744; doi:10.3389/fpubh.2023.1069906)
Supplement: Supplementary file 1 [file Table_1.DOCX]

Online Resource 3. Twenty-one differential metabolites in the two groups

| Sample Identification | P_(n)_^a^ | P_(e)_ ^b^ |
| --- | --- | --- |
| lysoPC a C16:1 | 0.003 | 0.021 |
| TG(22:6_34:1) | 0.062 | 0.018 |
| lysoPC a C18:0 | 0.001 | 0.066 |
| CE(20:5) | 0.268 | 0.001 |
| PC aa C36:6 | 0.116 | 0.004 |
| TG(22:6_34:2) | 0.059 | 0.030 |
| GABA | 0.324 | 0.000 |
| PC ae C44:6 | 0.204 | 0.006 |
| Arginine | 0.017 | 0.013 |
| lysoPC a C16:0 | 0.001 | 0.086 |
| PC aa C36:5 | 0.165 | 0.001 |
| SM C22:3 | 0.245 | 0.001 |
| lysoPC a C17:0 | 0.061 | 0.044 |
| GCDCA | 0.019 | 0.021 |
| PC aa C40:6 | 0.281 | 0.009 |
| TG(16:0_40:7) | 0.037 | 0.053 |
| TG(20:5_34:1) | 0.025 | 0.004 |
| Betaine | 0.030 | 0.169 |
| TMAO | 0.192 | 0.021 |
| PC ae C44:5 | 0.107 | 0.096 |
| Glutamine | 0.025 | 0.252 |

^a^P_(n)_: never smoker; ^b^P_(e)_: ever smoker. GABA: γ-aminobutyric acid, GCDCA: glycochenodeoxycholic acid, TMAO: trimethylamine N-oxide.
