# Supplementary figures and images for: Outdoor particulate matter exposure affects metabolome in chronic obstructive pulmonary disease: Preliminary study
Source: Front Public Health. 2023 Mar 21;11:1069906. doi: 10.3389/fpubh.2023.1069906 (PMC10070744; doi:10.3389/fpubh.2023.1069906)

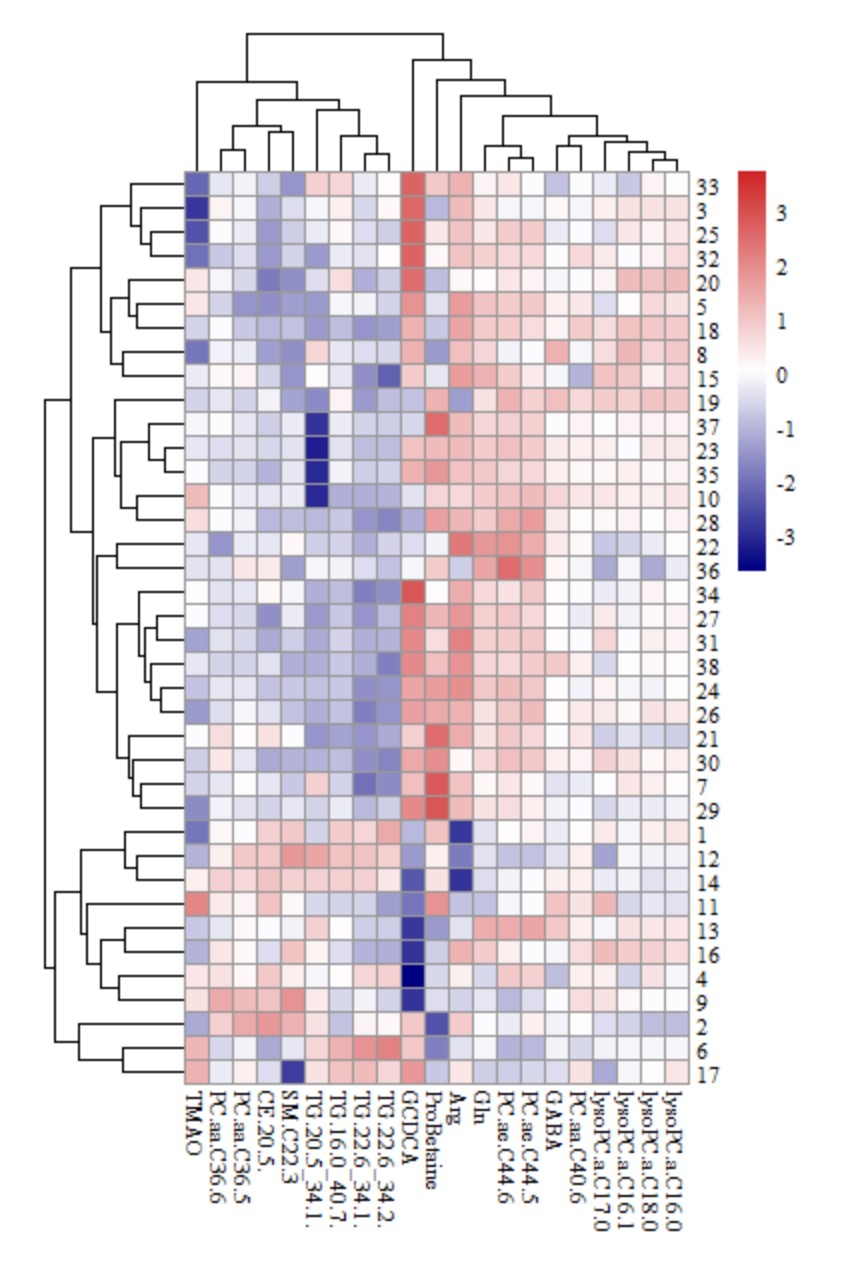

Supplement: Supplementary file 2 [file Image_1.JPEG]

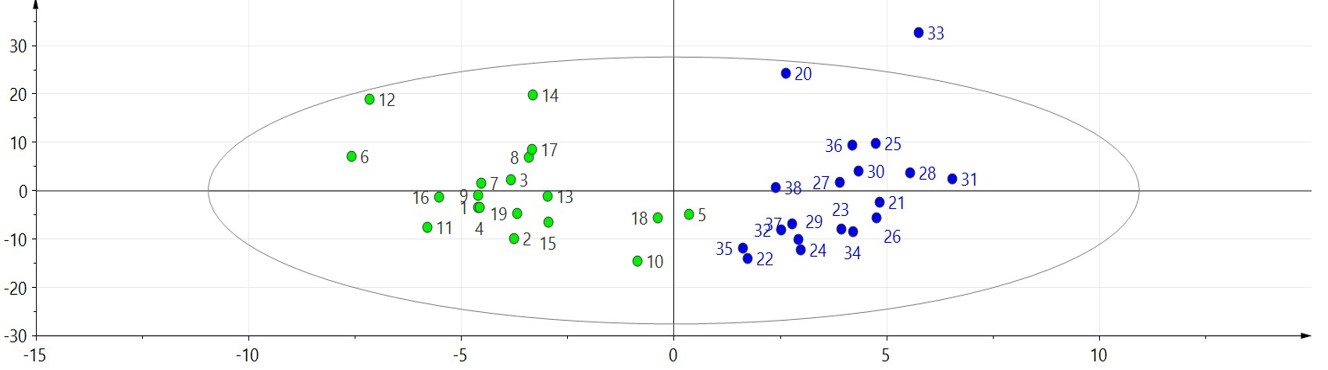

Supplement: Supplementary file 3 [file Image_2.JPEG]
